# Supplementary material for: Antiferromagnetic textures in BiFeO3 controlled by strain and electric field
Source: Nat Commun. 2020 Apr 6;11:1704. doi: 10.1038/s41467-020-15501-8 (PMC7136242; doi:10.1038/s41467-020-15501-8)
Supplement: Supplementary file 1 — Supplementary Information [file 41467_2020_15501_MOESM1_ESM.pdf]

Supplementary Information for

Antiferromagnetic textures in $BiFeO_3$ controlled by strain and electric field

A. Haykal[1*], J. Fischer[2*], W. Akhtar[1***], J.-Y. Chauleau[3], D. Sando[4], A. Finco[1], F. Godel[2], Y.A. Birkhölzer[5] C. Carrétéro[2], N. Jaouen[6], M. Bibes[2], M. Viret[3], S. Fusil[2,7**], V. Jacques[1], V. Garcia[2]

[1]*Laboratoire Charles Coulomb, Université de Montpellier and CNRS, 34095 Montpellier, France*

[2]*Unité Mixte de Physique, CNRS, Thales, Université Paris-Saclay, 91767 Palaiseau, France*

[3]*SPEC, CEA, CNRS, Université Paris-Saclay, 91191 Gif-sur-Yvette, France*

[4]*School of Materials Science and Engineering, University of New South Wales, Sydney 2052, Australia*

[5]*Department of Inorganic Materials Science, Faculty of Science and Technology and MESA+ Institute for Nanotechnology, University of Twente, P.O. Box 217, 7500 AE Enschede, The Netherlands*

[6]*Synchrotron SOLEIL, 91192 Gif-sur-Yvette, France*

[7]*Université d'Evry, Université Paris-Saclay, Evry, France*

*These authors contributed equally to this work

**e-mail: stephane.fusil@cnrs-thales.fr

***Now at Department of Physics, JMI, Central University, New Delhi, India

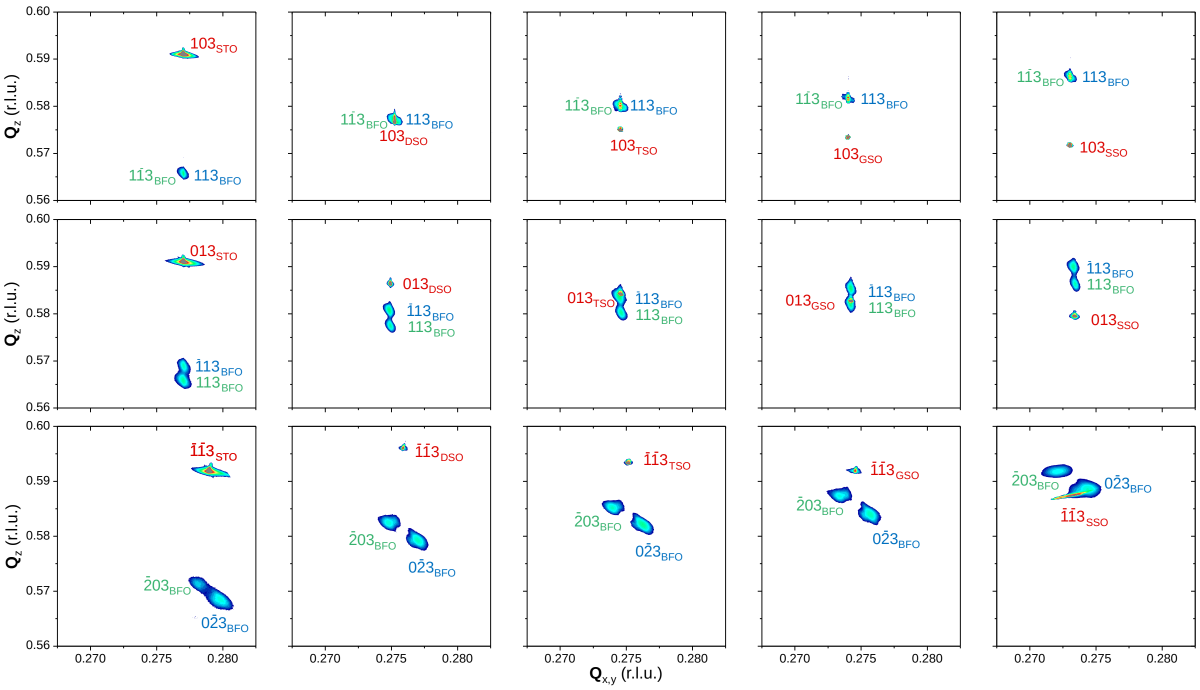

Supplementary Figure 1. Reciprocal space maps on (103), (013), $(\bar{1}\bar{1}3)$ substrate peaks. BFO, STO, DSO, TSO, GSO and SSO stand for $BiFeO_3$, $SrTiO_3$, $DyScO_3$, $TbScO_3$, $GdScO_3$ and $SmScO_3$, respectively. While the red colour label is assigned to the substrate peaks, the green and blue colour labels stand for the two elastic domains of the $BiFeO_3$ thin films. All the films display similar structural properties despite the large strain variations from $SrTiO_3$ to $SmScO_3$. The r. l. u. units of the in-plane and out-of-plane wavevectors, $\mathbf{Q}_{x,y}$ and $\mathbf{Q}_z$, respectively, stand for reciprocal lattice units.

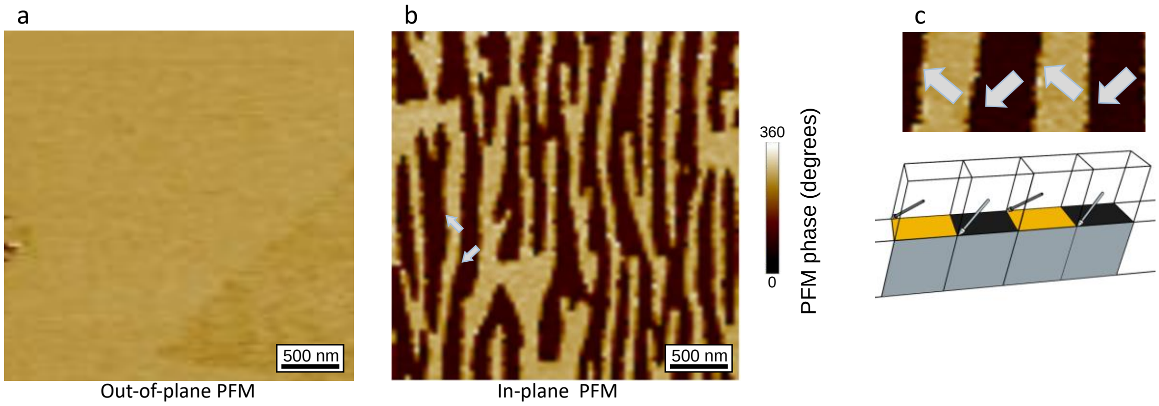

Supplementary Figure 2. Striped ferroelectric domains in $BiFeO_3$ with 71-degree domain walls. a, Out-of-plane PFM phase image of a $BiFeO_3$ film grown on $TbScO_3$(110). The homogeneous bright signal indicates a downward polarisation. **b**, Corresponding in-plane PFM phase image. The striped-domain structure corresponds to two polarisation variants (grey arrows). **c**, Sketch of the 71-degree domain wall structure.

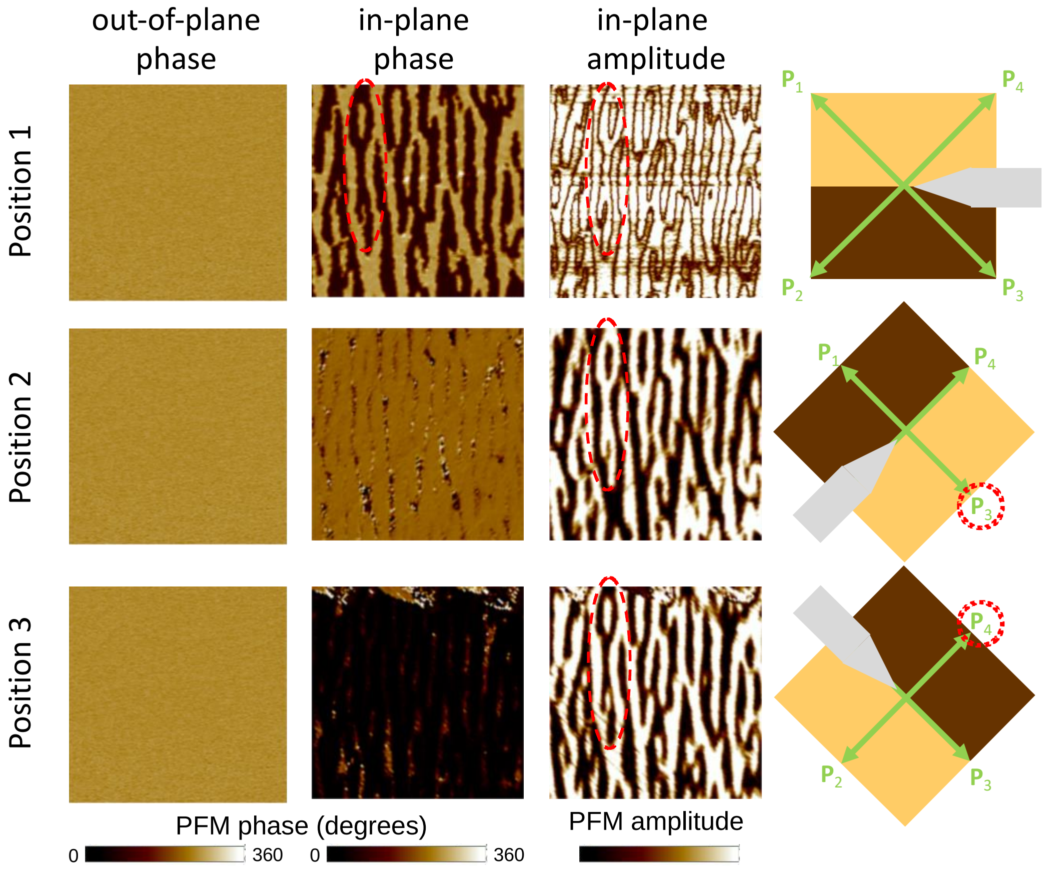

Supplementary Figure 3. Determining the 71-degree domain walls structure with vectorial piezoresponse force microscopy. Illustration for $BiFeO_3$ thin films grown on $SrRuO_3/DyScO_3(110)$. In position 1, the cantilever is perpendicular to the ferroelectric stripes. The out-of-plane PFM phase is homogeneous and bright, indicating only downward polarisation variants. The in-plane PFM phase shows alternating bright and dark domains with equal amplitudes. As sketched on the right panel, this could correspond to ($\mathbf{P}_1$, $\mathbf{P}_4$) variants in the bright regions and ($\mathbf{P}_2$, $\mathbf{P}_3$) variants in the dark regions. In position 2, the cantilever is parallel to ($\mathbf{P}_2$, $\mathbf{P}_4$), thus these two variants do not respond. The in-plane PFM phase and amplitude show that only one family of domains responds and its phase signal is thus bright. This signal corresponds to the $\mathbf{P}_3$ variant (right of the cantilever). In position 3, the cantilever is parallel to ($\mathbf{P}_1$, $\mathbf{P}_3$). The in-plane PFM phase and amplitude show that only one family of domains responds and its phase signal is therefore dark. This signal corresponds to the $\mathbf{P}_4$ variant (left of the cantilever) and the $\mathbf{P}_3$ variant does not respond as it is parallel to the cantilever. Putting all this information together allows us to conclude that the striped-domain structure then corresponds to alternated $\mathbf{P}_3$ and $\mathbf{P}_4$ domains with 71-degree domain walls. All the PFM images are 2.5×2.5 μm^2. The dashed red line emphasizes the complementarity between each signal in the three different positions.

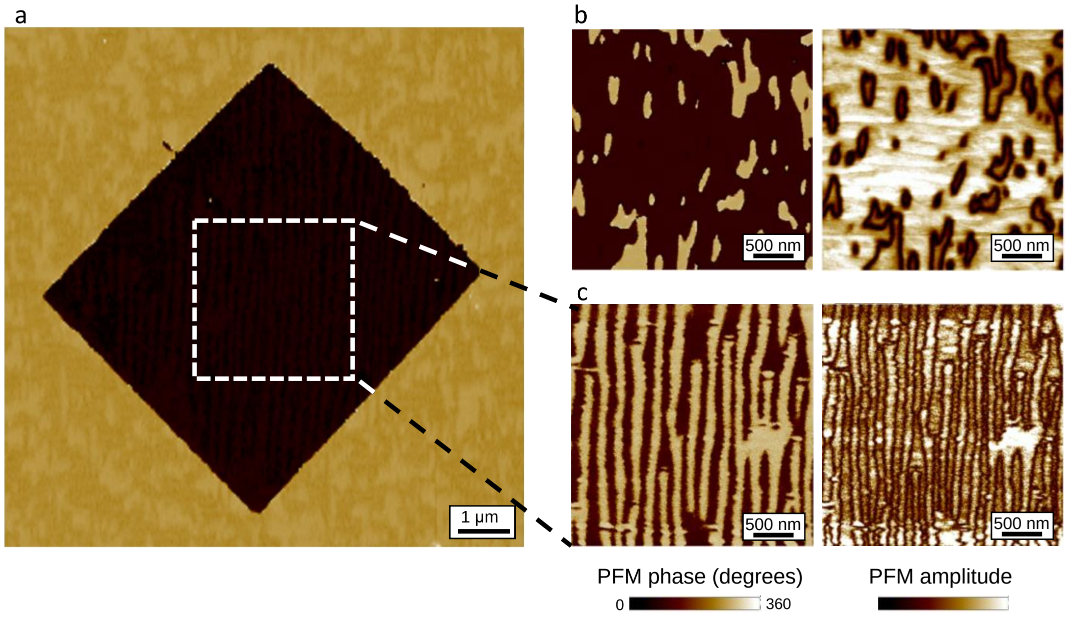

Supplementary Figure 4. Artificial stripes designed by PFM on $BiFeO_3$ thin films grown on $SrTiO_3$. a, Out-of-plane PFM phase change from domains pointing downwards (bright contrast) to domains pointing upwards (dark contrast). **b**,**c**, This writing scheme is accompanied by a change in the arrangement of the in-plane polarisation variants from the native mosaic-like pattern (**b**) to a stripe-domain pattern (**c**).

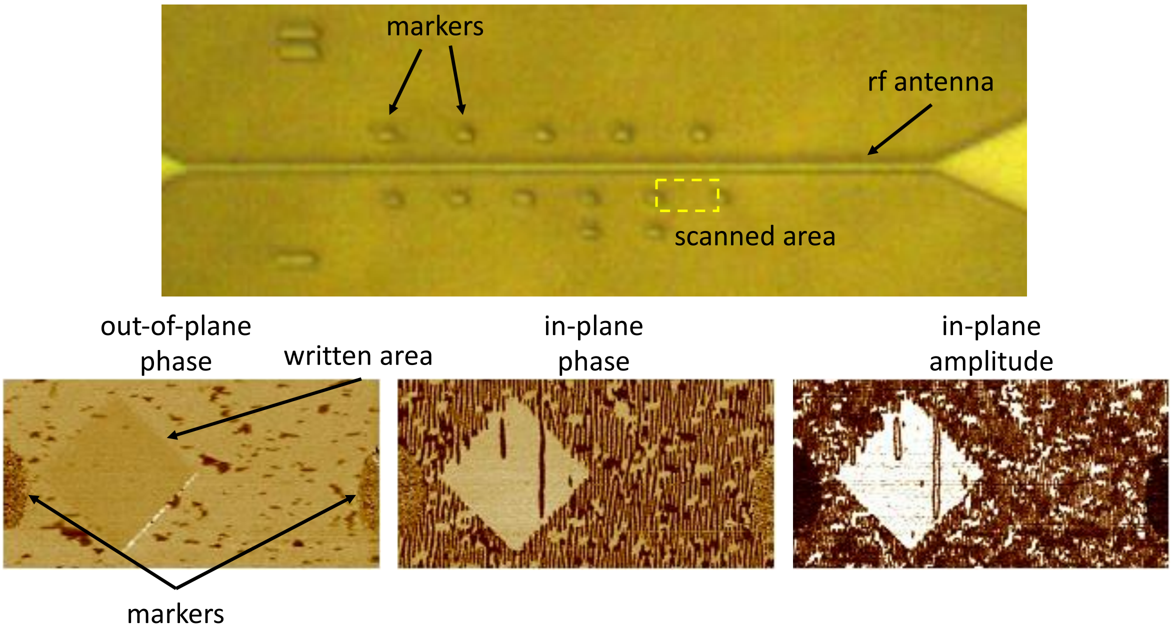

Supplementary Figure 5. Antenna and markers defined to spatially correlate PFM and NV imaging. Antenna and markers defined by laser lithography on the $BiFeO_3$ samples. (top) Optical microscope image. (bottom) 17 × 8.5 μm^2 PFM images in the scanned area defined by the dashed yellow square.

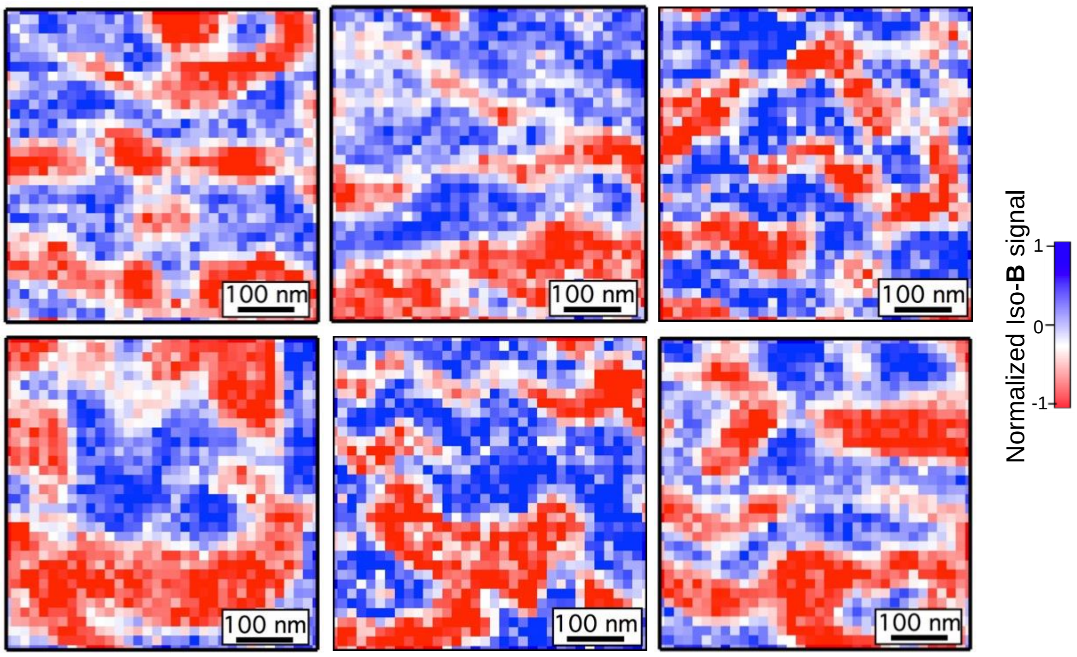

Supplementary Figure 6. Magnetic textures in $BiFeO_3$ thin films grown on $SmScO_3$. NV magnetometry images at different locations of the $BiFeO_3$ film grown on $SrRuO_3$/$SmScO_3$(110).

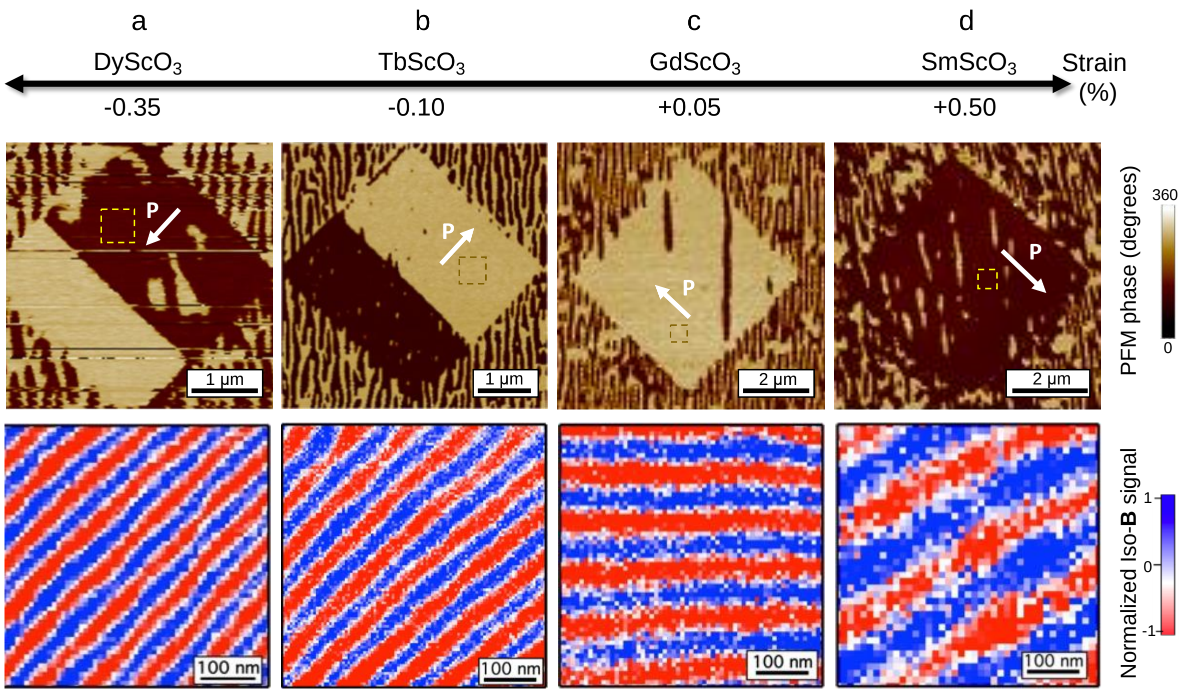

Supplementary Figure 7. Single ferroelectric domains and the corresponding magnetic textures. a-d (top) In-plane PFM phase images of written areas and (bottom) corresponding NV images for $BiFeO_3$ films grown on (**a**) $DyScO_3$, (**b**) $TbScO_3$, (**c**) $GdScO_3$, and (**d**) $SmScO_3$ substrates. The dashed squares in the PFM images show the sizes of the corresponding NV images.

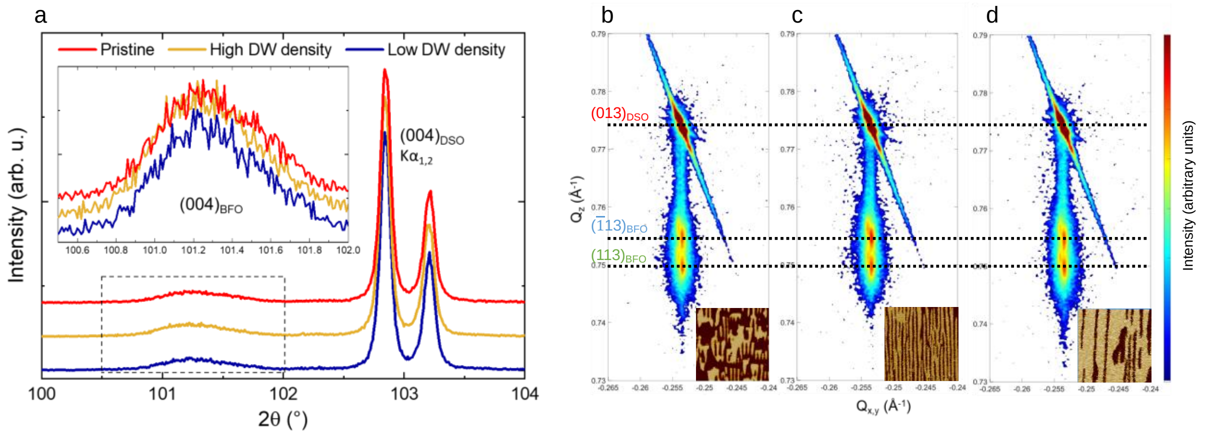

Supplementary Figure 8. Microdiffraction on pristine and written areas of a $BiFeO_3$ thin film grown on $DyScO_3$. a, $2\theta - \omega$ XRD scans of the (004) peaks and **b**-**d**, RSMs around the (013) substrate peak collected from a pristine area (**b**), a written area with high domain wall (DW) density (**c**), and a written area with low domain wall density (**d**). The dotted horizontal lines are guides to the eye to aid comparison. The insets in panels (**b-d**) show 4×4 μm^2 in-plane PFM phase images of the areas measured by microdiffraction. All three areas show identical structural properties despite the large variations in domain configurations, ruling out strain differences between artificially-written and as-grown striped-domains.
